# Melatonin promotes osteoblast differentiation by regulating Osterix protein stability and expression

**DOI:** 10.1038/s41598-017-06304-x

**Published:** 2017-07-18

**Authors:** Younho Han, Young-Mi Kim, Hyung Sik Kim, Kwang Youl Lee

**Affiliations:** 1 College of Pharmacy & Research Institute of Drug Development, Chonnam National University, Gwangju, Republic of Korea; 2 Department of Pharmacy, College of Pharmacy and Institute of Pharmaceutical Science and Technology, Hanyang University, Ansan, Gyeonggi-do 15588 Republic of Korea; 3 Division of Toxicology, College of Pharmacy, Sungkyunkwan University, Seoburo 2066, Suwon, Gyeonggi-Do 440-746 Republic of Korea

## Abstract

Although the biological role of melatonin in osteogenic differentiation has been suggested, the mechanism of osteoblast differentiation remains unclear. Thus, the present study investigated the underlying molecular mechanisms based on osteoblast-specific transcription factors. We found that melatonin enhanced BMP-4-induced osteogenic differentiation and increased the expression of osteogenic markers, especially Osterix, which is an essential transcription factor for the differentiation of preosteoblasts into mature osteoblasts in the late stage of osteoblast differentiation. Melatonin treatment increased the expression of Osterix during osteoblast differentiation and stabilized its expression by the inhibition of ubiquitin-proteasome-mediated degradation of Osterix, leading to up-regulated Osterix transcriptional activity on the osteogenic promoter and promoting alkaline phosphatase activity and bone mineralization. Furthermore, treatment with protein kinase A (PKA) inhibitor H89 and protein kinase C (PKC) inhibitor Go6976 blocked the melatonin-induced transcriptional activity and phosphorylation of Osterix, indicating that melatonin regulates Osterix expression via the PKA and PKC signaling pathways. Overall, these findings suggest that melatonin directly regulates the late stage of osteoblast differentiation by enhancing Osterix expression; this provides further evidence of melatonin as a potent agent for treating osteoporosis.

## Introduction

Osteoblasts are bone-forming cells derived from mesenchymal stem cells. They differentiate from fibroblasts during skeletal development to function in the formation of bone tissue^1^. As an effective therapeutic approach, anabolic agents targeting the stimulation of osteoblastic differentiation can improve trabecular bone microarchitecture and restore bone loss through the inhibition of bone resorption^2, 3^. Various signaling pathways, including Wnt, bone morphogenetic protein (BMP), Hedgehog, Notch, and fibroblast growth factors have been implicated in the regulation of osteoblast differentiation^4^. Among them, BMPs including BMP-2 and −4 appear to have an important role in the differentiation of mesenchymal stem cells into osteoblasts via the activation of transcription factors, such as Runx2/core binding factor a1 (Cbfa1) and Sp7/Osterix^5^.

Melatonin is a hormone involved in regulating circadian rhythms, including initiation and sustenance of sleep^6, 7^. Secretion of melatonin is enhanced in darkness and repressed by light; this process is regulated by the suprachiasmatic nucleus of the hypothalamus^8^. Melatonin is a remarkably conserved molecule with diverse physiological and pathophysiological functions, including regulation of circadian rhythms, immune and antioxidant defense, tumor growth inhibition, and reproduction control^9–14^. Additionally, accumulating evidence from previous experiments performed *in vitro* and *in vivo* has demonstrated the possible function of melatonin in bone formation and development^15–17^. During osteoblastic differentiation, treatment with melatonin resulted in an increased expression of alkaline phosphatase (ALP), bone sialoprotein (BSP), and osteocalcin (OC) genes, which ultimately promoted matrix mineralization in MC3T3-E1 cells^18^. Moreover, an intraperitoneal administration of melatonin slightly enhanced new cortical bone formation in the femurs of mice^19^. In addition, melatonin promoted mineralization and osteoblastic differentiation by enhancing the expression of Runx2, which is a key transcription factor in the early stage of osteogenic differentiation, via the mitogen-activated protein kinase (MAPK) signaling pathway^20^.

Although many previous studies have elucidated the mechanism of melatonin in osteogenic differentiation, its precise effect on the differentiation of preosteoblasts into mature osteoblasts remains to be investigated. In this study, we aimed to determine whether melatonin regulates the late stage of osteogenic differentiation.

## Results

### Melatonin enhances the BMP-4-induced osteogenic differentiation

**Figure 1:**
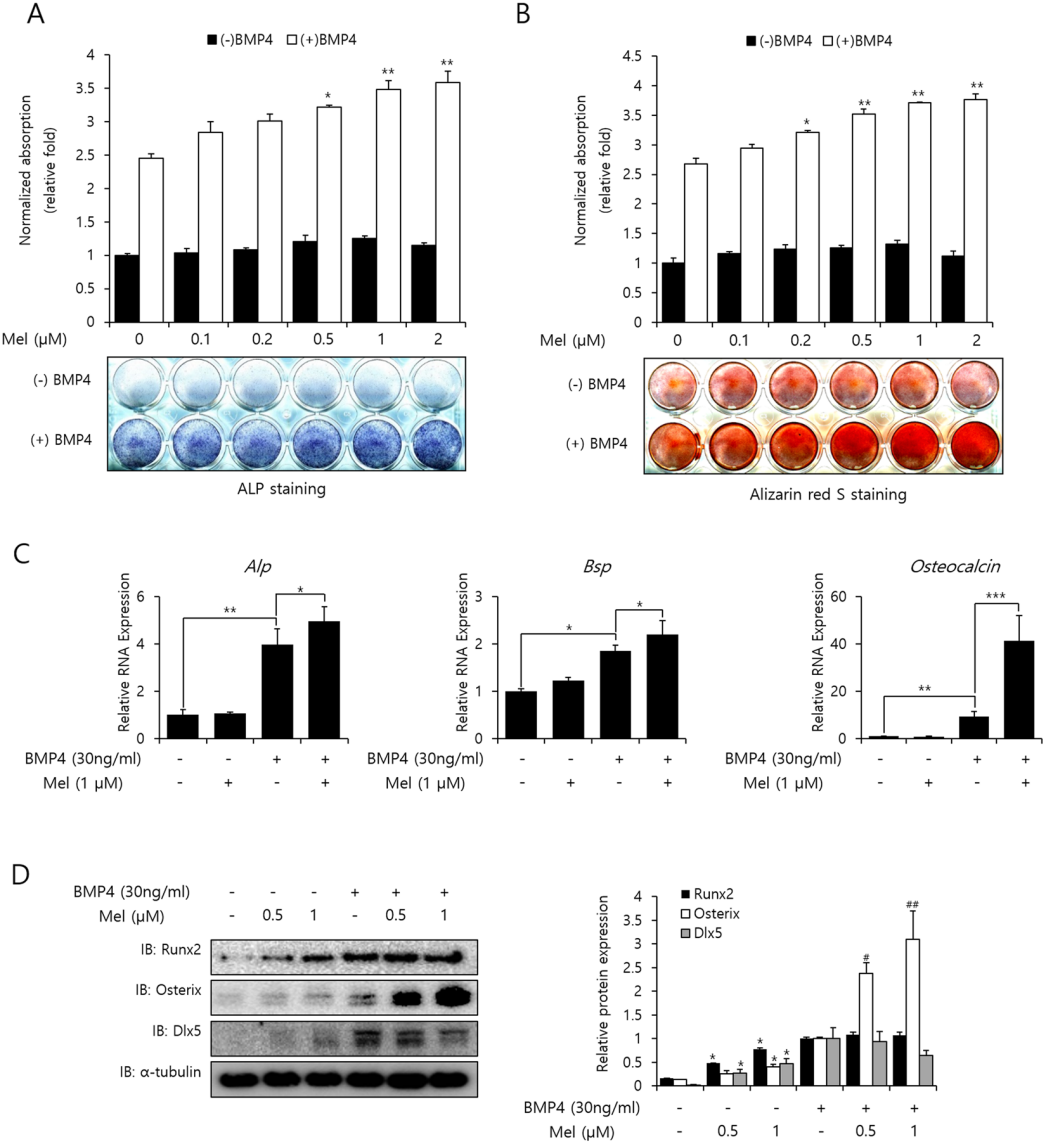
Melatonin promotes BMP-4-induced osteogenic differentiation. (**A** and **B**) C2C12 cells were treated with BMP-4 (30 ng/mL) and exposed to various concentrations of melatonin for 3 days (ALP staining) or 10 days (ARS staining). Quantification of ALP and ARS staining was performed at an absorbance of 480 and 405 nm, respectively. *P < *0*.*05*, **P < *0*.*01* compared with BMP-4-treated group. (**C**) C2C12 cells were treated with BMP-4 (30 ng/mL) and exposed to melatonin (1 µM) for 3 days. mRNA expression levels of the osteoblast-specific markers, ALP, BSP, and OC were determined by real-time PCR and normalized to GAPDH. *P < *0*.*05*, **P < *0*.*01*, ***P < *0*.*001* (**D**) C2C12 cells were treated with BMP-4 (30 ng/mL) and exposed to melatonin (0.5, or 1 µM) for 3 days. The protein expression levels of Runx2, Osterix, and Dlx5 were confirmed by immunoblotting. α-tubulin was used as a loading control. (Full-length blots with high contrast of each tested protein are reported in Supplementary Fig. S7). The ratio of relative protein expression of Runx2, Osterix, and Dlx5 was normalized to the BMP-4-treated group. *P < *0*.*05* compared with control group. ^#^ *P* < *0*.*05*, ^*##*^ *P* < *0*.*01* compared with BMP-4–treated group. Statistical analysis by one-way ANOVA (**A**,**B**, and **D**) or two-way ANOVA (**C**). Data are representative of three independent experiments [mean ± SD of two replicates in **A**,**B** and **D**, and three replicates in C].

ALP staining was conducted to explore the effect of melatonin on osteoblast differentiation. The ALP activity of C2C12 cells was increased significantly in the presence of BMP-4, and further increased by melatonin treatment in a dose-dependent manner (Fig. 1A). Additionally, mineralization of these cells was examined by ARS staining. Results showed that mineralization was increased in the melatonin-treated cells, as seen by dense red staining in these cells compared with the untreated control cells (Fig. 1B). Melatonin did not exhibit significant effects on cell proliferation at the concentrations used after 48 and 72 h of treatment in C2C12 cells (Fig. 1S). The gene expression profiles of osteogenic markers, including ALP, BSP, and OC were investigated by quantitative PCR (qPCR) after 3 days of treatment with BMP-4 and melatonin. Melatonin significantly increased the mRNA expression levels of ALP, BSP, and OC (Fig. 1C). Protein levels of the osteoblastic transcription factors, including Runx2, Osterix, and Dlx5 were detected by immunoblotting. As shown in Fig. 1D, in the absence of BMP-4 induction, melatonin treatment slightly enhanced the expression of Runx2, Osterix, and Dlx5. Meanwhile, Osterix expression was significantly up-regulated with 1 μM melatonin by about 3-fold upon the stimulation of BMP-4.

### Melatonin promotes the osteogenic transcriptional activity

**Figure 2:**
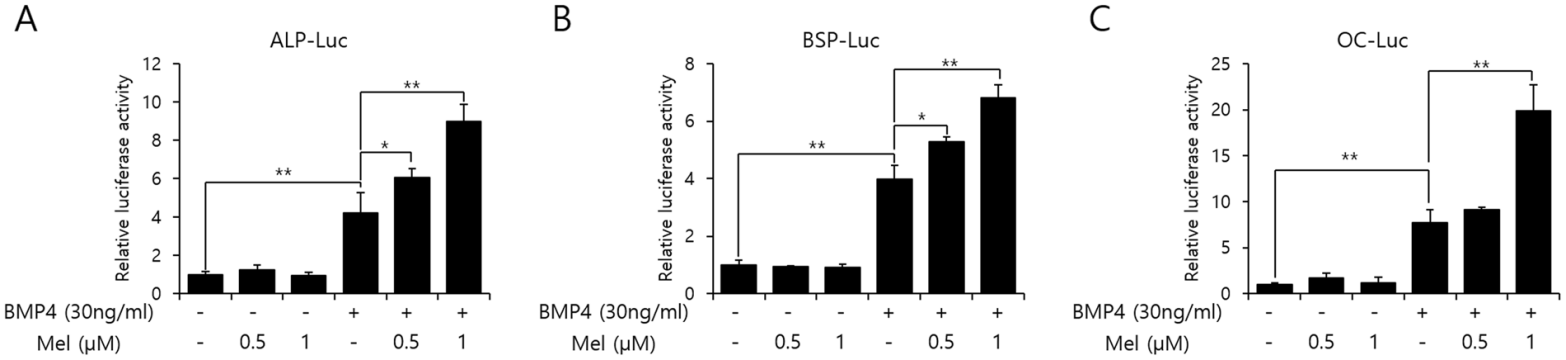
Melatonin enhances BMP-4-induced transcriptional activity. (**A**–**C**) C2C12 cells were transfected with pCMV-β-gal (0.1 µg), luciferase reporters [(**A**) ALP-Luc, (**B**) BSP-Luc, or (**C**) OC-Luc; 0.3 µg] and exposed to melatonin (0.5, or 1 µM). Luciferase activities were measured. Statistical analysis by two-way ANOVA. *P < *0*.*05*, **P < *0*.*01*. Data are representative of three independent experiments [mean ± SD of three replicates].

To elucidate if melatonin has any effect on BMP-4-induced transcriptional activity, we performed luciferase assay using ALP, BSP, and OC promoter. More specifically, the transcriptional activity of ALP, BSP, and OC was up-regulated at similar concentrations of melatonin (Fig. 2A–C), suggesting that melatonin is capable of consistently stimulating the BMP-4-induced osteoblast differentiation of C2C12 cells.

### Melatonin up-regulates the Osterix stabilization

**Figure 3:**
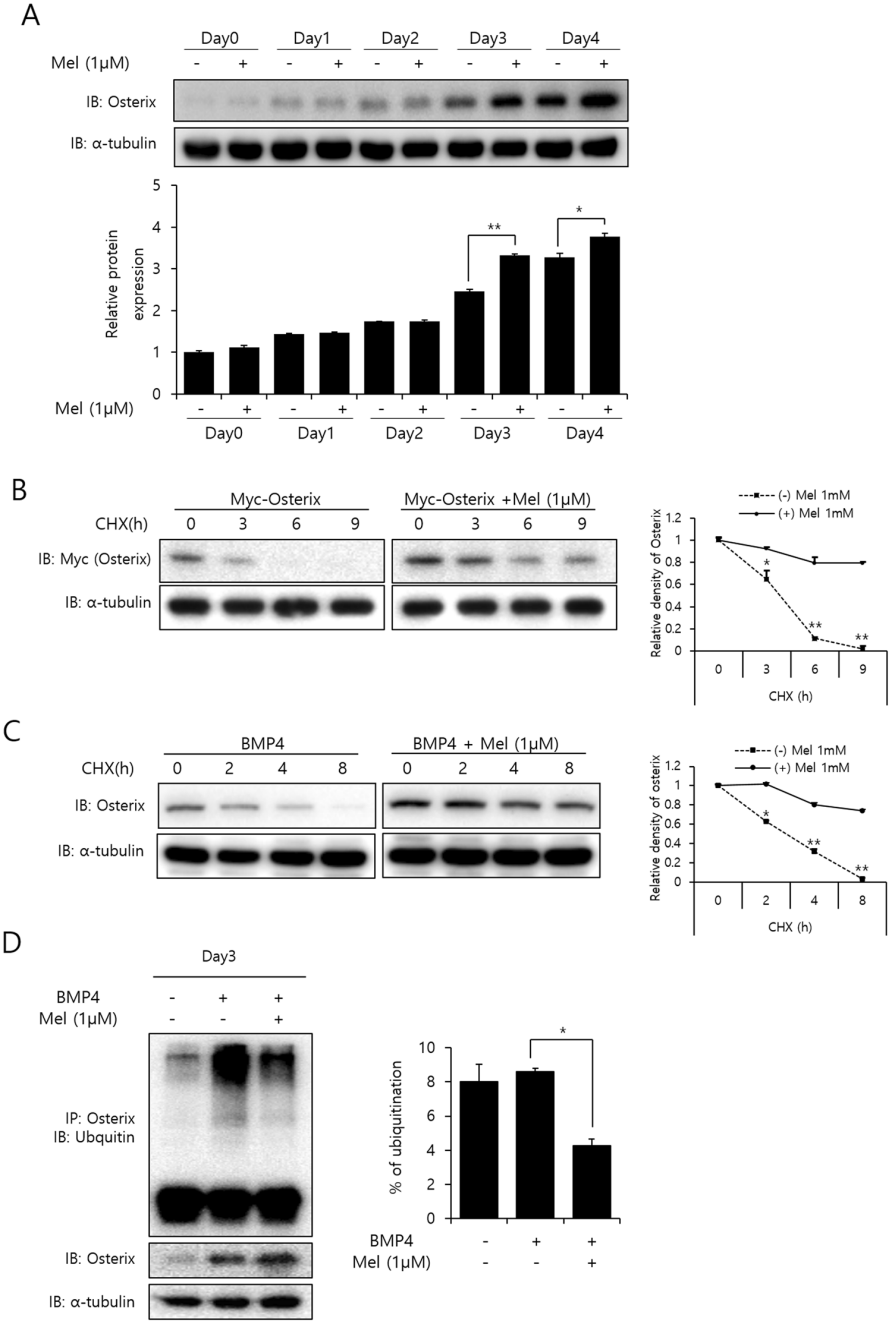
Melatonin stabilizes the Osterix expression. (**A**) C2C12 cells were treated with BMP-4 (30 ng/mL) and exposed to melatonin (1 µM) for the indicated time points. The protein expression of Osterix was confirmed by immunoblotting. α-tubulin was used as a loading control. (Full-length blots with high contrast of each tested protein are reported in Supplementary Fig. S8). The ratio of relative protein expression of Osterix on indicated days was normalized to the protein expression of Osterix on day 0 in the absence of melatonin. *P < *0*.*05*, **P < *0*.*01*. (**B**) C2C12 cells were transfected with Myc-Osterix (1 µg) and then treated with melatonin (1 µM). After 48 h, cells were treated with CHX (40 μg/mL) for 0–9 h, and the cell lysates were subjected to immunoblotting. (Full-length blots with high contrast of each tested protein are reported in Supplementary Fig. S9). (**C**) C2C12 cells were treated with BMP-4 (30 ng/mL) and/or melatonin (1 µM) for 3 days. Cells were treated with CHX (40 μg/mL) for 0–8 h and the cell lysates were subjected to immunoblotting. (Full-length blots with high contrast of each tested protein are reported in Supplementary Fig. S10). The intensities of Osterix bands were measured by the image software, Multi Gauge V3.0 (FUJIFILM). The expression levels of Osterix in CHX-untreated cells (0 h) were set to 100%. *P < *0*.*05*, **P < *0*.*01* compared with melatonin-non treated group. (**D**) C2C12 cells were treated with BMP-4 (30 ng/mL) and/or melatonin (1 µM) for 3 days and the cells were treated with proteasome inhibitor, MG-132 (10 mM) for 4 h. Osterix immunoprecipitates (IPs, anti-Osterix) were analyzed using ubiquitin immuneblot (IB, anti-ubiquitin). The level of Osterix in cell lysates has been shown (middle panel). α-tubulin was used as the loading control (bottom panel). (Full-length blots with high contrast of each tested protein are reported in Supplementary Fig. S11). Relative ubiquitination was measured by densitometry after normalization for the amount of Osterix. *P < *0*.*05*. Statistical analysis by one-way ANOVA (**A**) or two-way ANOVA (**B**,**D**). Data are representative of three independent experiments [mean ± SD of two replicates in **A**–**D**].

Since melatonin considerably increased Osterix expression at the protein level, it was suspected that melatonin could regulate degradation of Osterix. We first determined the Osterix expression during the course of osteoblast differentiation, in the absence or presence of melatonin. Cellular Osterix expression was continuously increased from the onset of osteoblast differentiation, and treatment with melatonin further enhanced its expression, especially on day 3 and 4 (Fig. 3A). To investigate whether ubiquitin-proteasome-mediated degradation contributed to the regulation of melatonin on Osterix abundance, we compared the protein degradation half-life of Osterix in the absence or presence of melatonin. As shown in Fig. 3B and C, exogenous and endogenous Osterix were degraded with a similar half-life (*t½* ≃ 2–3 h). Incubation of cells with melatonin significantly slowed the rate of Osterix degradation (*t½* > 8–9 h). In addition, we explored if melatonin regulates Osterix polyubiquitination, which is an essential process for its proteasomal degradation, and found that melatonin decreased the amount of Osterix modified with ubiquitin (Fig. 3D).

### Melatonin stimulates osteogenic activity of Osterix

**Figure 4:**
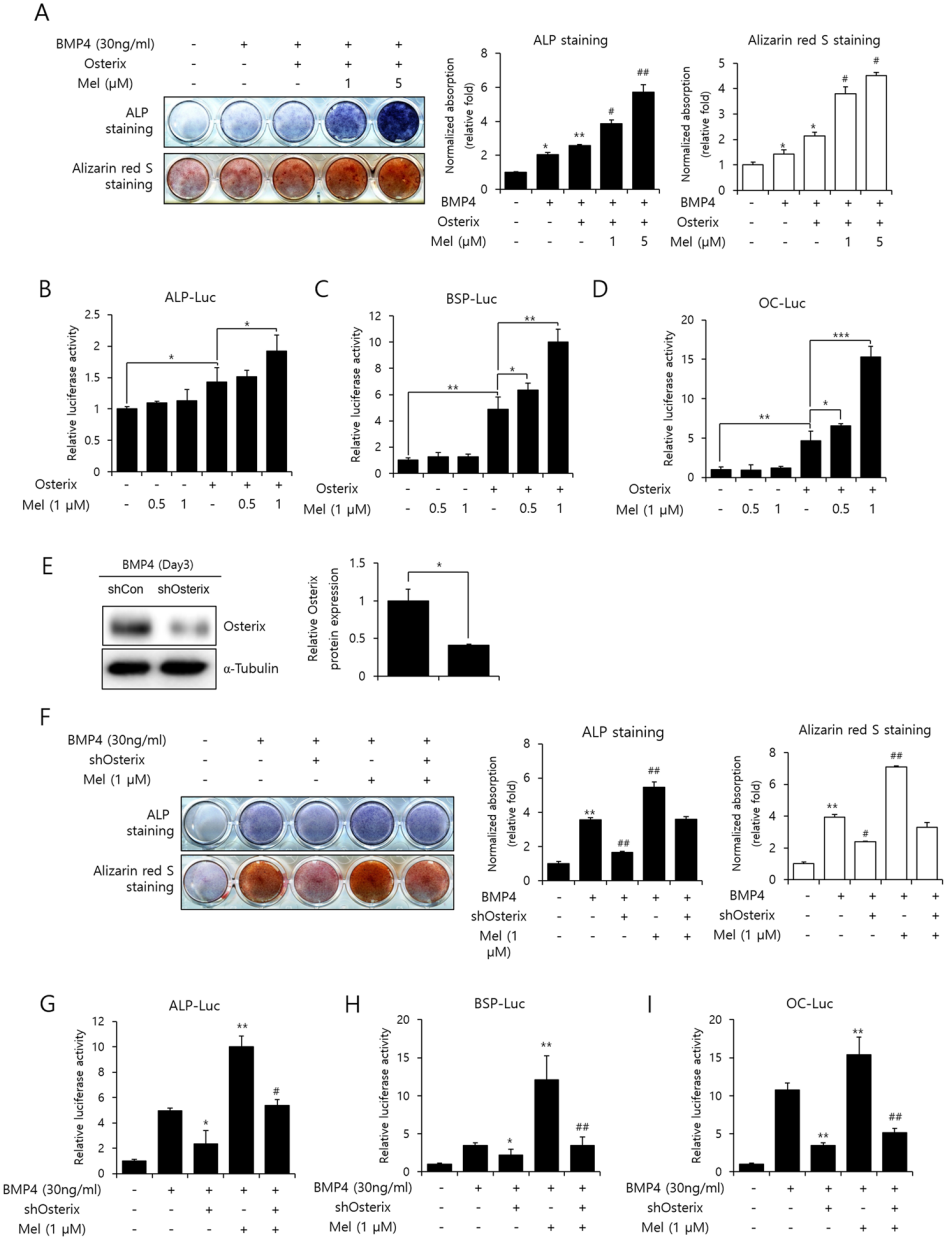
Melatonin stimulates osteoblast differentiation by Osterix activation. (**A**) C2C12 cells were transfected with Myc-Osterix (1 µg) and then treated with BMP-4 (30 ng/mL) and melatonin (1 or 5 µM). The extent of ALP activity and mineralization was evaluated using ALP or ARS staining for 3 or 10 days, respectively. *P < *0*.*05*, **P < *0*.*01* compared with control group. ^*#*^ *P* < *0*.*05*, ^*##*^ *P* < *0*.*01* compared with BMP-4–treated and Osterix-transfected group (**B**–**D**) C2C12 cells were transfected with pCMV-β-gal (0.1 µg), Myc-Osterix (0.3 µg), and luciferase reporter [(**B**) ALP-Luc, (**C**) BSP-Luc, or (**D**) OC-Luc; 0.3 µg], and exposed to melatonin (0.1, 0.5, or 1 µM). Luciferase activities were measured. *P < *0*.*05*, **P < *0*.*01*, ***P < *0*.*001*. (**E**) Knockdown efficiency of Osterix expression levels upon the stimulation of BMP-4 (30 ng/mL) on day 3 was quantified by Western blot analysis with anti-Osterix and anti-α-Tubulin antibodies. (Full-length blots with high contrast of each tested protein are reported in Supplementary Fig. S12). The ratio of relative protein expression of Osterix was normalized to shCon of Osterix. *P < *0*.*05*. (**F**) C2C12 cells were transfected with pSuper-Osterix (shOsterix) and then treated with BMP-4 (30 ng/mL) and melatonin (1 µM). The extent of ALP activity and mineralization was evaluated using ALP or ARS staining for 3 or 10 days, respectively. Quantification of ALP and ARS staining was performed at an absorbance of 480 and 405 nm, respectively. *P < *0*.*05*, **P < *0*.*01* compared with control group. ^*#*^ *P* < *0*.*05*, ^*##*^ *P* < *0*.*01* compared with BMP-4–treated group. (**G–I**) C2C12 cells were transfected with pCMV-β-gal (0.1 µg), shOsterix (0.3 µg), and luciferase reporter [(**G**) ALP-Luc, (H) BSP-Luc, or (I) OC-Luc; 0.3 µg], and exposed to melatonin (1 µM). Luciferase activities were measured. *P < *0*.*05*, **P < *0*.*01* compared with BMP-4-treated group. ^*#*^ *P* < *0*.*05*, ^*##*^ *P* < *0*.*01* compared with BMP-4- and melatonin-treated group. Statistical analysis by one-way ANOVA (**E**) or two-way ANOVA (**A**–**D** and **F**–**I**). Data are representative of three independent experiments [mean ± SD of two replicates in (**A** and **F**), and three replicates in **B**–**D** and **G**–**I**].

All of these results demonstrated that melatonin regulates Osterix protein stability via the ubiquitin-proteasome pathway, during osteoblast differentiation. The effect of melatonin on Osterix-induced osteoblast differentiation was assessed using ALP and ARS staining assays. The results showed that Osterix increased the BMP-4-induced ALP activity and matrix mineralization, and melatonin treatment further enhanced osteoblast differentiation (Fig. 4A). Next, we performed luciferase assay using ALP, BSP, and OC promoters containing reporter constructs in which luciferase expression could be induced by Osterix, with or without exposure to melatonin. The transcriptional activity was increased in response to Osterix overexpression, whereas culture of transfected cells in the presence of melatonin led to an up-regulation of Osterix promoter activity in a dose-dependent manner (Fig. 4B–D). In order to clarify whether the increased Osterix expression and activity were indeed mediated by melatonin, we examined the effects of melatonin after shRNA-mediated knockdown of Osterix mRNA (Fig. 4E). Even though melatonin increased BMP-4-induced ALP activity and mineralization, knockdown of Osterix entirely abolished the effects of melatonin (Fig. 4F). Additionally, the regulatory effect of melatonin on BMP-4-induced transcriptional activity was markedly inhibited by Osterix knockdown (Fig. 4G–I).

### Melatonin promotes Osterix activation through the PKA and PKC signaling pathway

**Figure 5:**
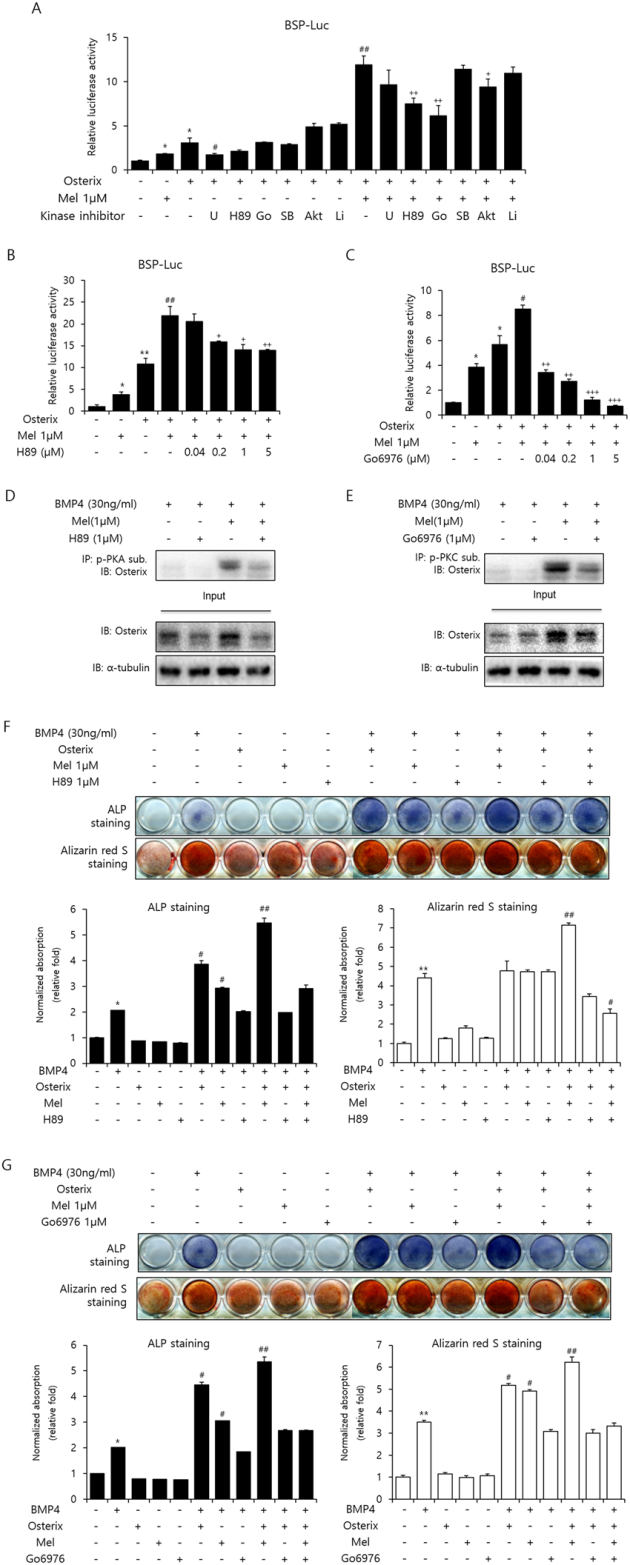
Melatonin regulates Osterix activation via PKA and PKC signaling pathway (**A**–**C**) C2C12 cells were transfected with pCMV-β-gal (0.1 µg), Myc-Osterix (0.3 µg), and luciferase reporter (BSP-Luc; 0.3 µg) with the indicated combinations of melatonin and various kinase inhibitors. U (U0126; 10 µM; MEK inhibitor), H89 (5 µM; PKA inhibitor), Go (Go6976; 5 µM; PKC inhibitor), SB (SB203580; 5 µM; p38 MAPK inhibitor), XI (5 µM; Akt inhibitor), and LiCl (1 mM; GSK3β inhibitor). Luciferase activities were measured. *P < *0*.*05*, **P < *0*.*01* compared with control group. ^*#*^ *P* < *0*.*05*, ^*##*^ *P* < *0*.*01* compared with Osterix-transfected group. ^*+*^ *P* < *0*.*05*, ^*++*^ *P* < *0*.*01*, ^*+++*^ *P* < *0*.*001* compared with Osterix-transfected and melatonin-treated group. (**D** and **E**) C2C12 cells were treated with BMP-4 (30 ng/mL) and/or melatonin (1 µM) for 3 days. Then, cells were incubated with H89 (**D**) or Go6976 (**E**) for 24 h. Osterix was immunoprecipitated with anti-phospho-(Ser/Thr) PKA substrate antibody (IP, p-PKA sub; **D**) or anti-phospho-PKC substrate antibody (IP, p-PKC sub; **E**), and analyzed by immunoblotting with an anti-Osterix antibody. (Full-length blots with high contrast of each tested protein are reported in Supplementary Fig. S13 and S14). (**F** and **G**) C2C12 myoblasts were treated with BMP-4 (30 ng/mL) and melatonin (1 µM), H89 (1 or 5 µM; **F**), or Go6976 (1 or 5 µM; **G**) for 3 days (ALP staining) or 10 days (ARS staining). Quantification of ALP and ARS staining was performed at an absorbance of 480 and 405 nm, respectively. *P < *0*.*05*, **P < *0*.*01* compared with control group. ^*#*^ *P* < *0*.*05*, ^*##*^ *P* < *0*.*01* compared with BMP-4–treated group. Statistical analysis by two-way ANOVA (**A**–**C**, **F**, and **G**). Data are representative of three independent experiments [mean ± SD of three replicates in A–C and two replicates in **F** and **G**].

It has been shown that the expression and transcriptional activity of Osterix are regulated by several extracellular signaling pathways, including ERK1/2, PKA, p38 MAPK, Akt, and GSK3β pathway^21–25^. To attenuate the ERK1/2 pathway, we used U0126, a specific inhibitor of MEK1/2, which is an upstream molecule of ERK1/2. H89, Go6976, SB203580, XI, and LiCl were used for the inhibition of PKA, PKC, p38 MAPK, Akt, and GSK3β pathways, respectively. Among the various kinase inhibitors, both H89 and Go6976 markedly attenuated the Osterix-induced transcriptional activity in the presence of melatonin (Fig. 5A–C). Based on this result, we speculated if melatonin modulates the PKA- or PKC-mediated phosphorylation of Osterix. To investigate this, Osterix expression was induced by BMP-4 treatment for 3 days. The phosphorylation of Osterix was assessed by immunoprecipitation with a phospho-PKA or -PKC substrate antibody, followed by the detection of Osterix antibody (Fig. 5D and E). The results revealed that the PKA- or PKC-induced phosphorylation of Osterix was increased by melatonin, but suppressed by H89 and Go6976, respectively. Additionally, the ALP and ARS staining results revealed that BMP-4-induced ALP activity and mineralization were enhanced by Osterix overexpression in the presence of melatonin, but significantly inhibited by H89 and Go6976 (Fig. 5F and G).

## Discussion

Over the past years, the possible effects of melatonin on bone formation have been frequently investigated, and melatonin has been considered as an alternative therapy for preventing and treating bone diseases^26, 27^. Moreover, as melatonin secretion slowly decreases with age and after menopause, the development of osteoporosis is accelerated in the elderly and postmenopausal women^28–30^. A recent study on human found that treatment with melatonin in postmenopausal women increases in bone mineral density (BMD) at the femoral neck^31^. Moreover, melatonin has a bone protective role in the oral cavity by reducing the periodontal inflammation, subsequently decreasing the oxidative stress^32^. One of the mechanisms by which melatonin regulates the bone environment is through its stimulatory effect on osteoblasts^33, 34^. A previous study revealed that melatonin enhances the differentiation of human adult mesenchymal stem cells into mature osteoblasts via MT2 melatonin receptors^35^, and that, during osteoblast differentiation, treatment with melatonin increases the expression levels of osteocalcin (OC), a late, highly specific osteoblast marker of bone formation, followed by enhanced bone mineralization of MC3T3-E1 cells^36^. Although it has been reported that melatonin promotes the expression of Runx2, a master transcription factor during the early stage of osteogenesis^37^, the precise mechanism by which it regulates the differentiation of preosteoblasts into mature osteoblasts remains unknown. Therefore, we investigated the underlying mechanisms and effects of melatonin on the late stage of osteoblast differentiation.

Osterix, a zinc-finger-containing transcription factor is required for bone formation and osteoblast differentiation. In Osterix-null mutant embryos, the mesenchyme and periosteum of the endochondral skeleton have been shown to be significantly decreased compared to levels common in other mesenchymal cells^38^. In addition, a previous study demonstrated that Osterix-null preosteoblasts are blocked from differentiating into mature osteoblasts; however, they express chondrogenic markers and can differentiate into both chondrocytes and osteoblasts^39^, indicating that Osterix is required for mature bone formation. The present study shows that melatonin increases Osterix expression as well as Osterix-mediated ALP activity, matrix mineralization, and transcriptional activity during induction of osteogenic differentiation (Figs 3A and 4). Moreover, ChIP (chromatin immunoprecipitation) analysis revealed that occupancy of Osterix at the promoter of the BSP gene was enhanced by melatonin treatment (Fig. S2). As previously reported, although melatonin enhances the expression of Runx2 slightly, Osterix expression is more significantly affected by melatonin and is correlated to a marked enhancement in the secretion of late osteogenic markers and in OC transcriptional activity, rather than that of ALP and BSP.

BMPs are the most important inducers and stimulators of osteoblast differentiation and play significant roles in the process of bone formation through SMAD-dependent pathways^40^. There are evidences that activation of the BMP signaling pathway induces Osterix expression^41^, and that melatonin not only enhances osteoblastic differentiation via the BMP signaling pathway, but also regulates the activity and expression of BMPs^42^, implying that melatonin could further enhance the BMP-induced expression and activity of Osterix. As expected, melatonin markedly increased the protein levels of Osterix induced by BMP in a dose- and time-dependent manner (Figs 1D and 3A) indicating that melatonin promotes osteoblast differentiation by up-regulating the BMP-induced Osterix expression. Additionally, ALP and ARS staining showed that ALP activity and matrix mineralization reached significance when the cells were treated with melatonin. In a previous study performed *in vivo*, melatonin administration increased the serum bone ALP activity and phosphorus and calcium levels, subsequently improved the bone mineral density (BMD) in ovariectomized rats^43^. Overall, these results indicate that melatonin can promote bone anabolic effect by up-regulating the osteoblastic functions.

It has been reported that Osterix expression and stability are regulated by ubiquitination-mediated proteasomal degradation pathways during osteoblast differentiation^44^. The interaction between melatonin and the ubiquitin-proteasome system has been shown in mitochondria, brown fat, and oxidative stress^45^. Moreover, the suppressive effect of melatonin on ubiquitin-proteasome-mediated protein degradation regulates various osteoblastic markers and maintains a balance between bone formation and resorption^46^. With regard to the mechanisms underlying the regulation of Osterix expression by melatonin, we considered the possibility that this may be mediated via inhibition of the ubiquitin-proteasome degradation pathway. In the current study, melatonin stabilized the expression and prolonged the half-life of Osterix, mediated by the downregulation of Osterix polyubiquitination (Fig. 3). In our previous report, the E3 ligases, Cbl-b and c-Cbl, induced Osterix ubiquitination and degraded the expression of Osterix^47^. A study conducted by Lian *et al*. reported that melatonin treatment downregulates ubiquitination, mediated by the E3 ubiquitin ligase SMURF1, and degradation of SMAD1, leading to osteogenesis of mesenchymal stem cells by sustaining the BMP-SMAD1 signaling cascade^48^. Consistently, our additional results showed that melatonin treatment slightly enhances the BMP4-induced SMAD signaling pathway (Fig. S3). Additionally, melatonin treatment partially recovered the Osterix expression degraded by SMURF1, Cbl-b, and c-Cbl through the downregulation of their expression (Fig. S4) and melatonin further increased the Osterix expression up-regulated by co-expression of SMAD1/5 (Fig. S5). Therefore, there is a possibility that melatonin could regulate the various E3 ligases-induced ubiquitination and degradation of Osterix, however, the exact manner in which melatonin regulates Osterix expression through the several E3 ligases still remains unknown.

In addition to ubiquitination, phosphorylation is an essential process in the modulation of Osterix expression, and there is close crosstalk between ubiquitination and phosphorylation^49–52^. Several lines of evidence suggest that melatonin modulates mesenchymal stem cell differentiation and completes mature osteoblast differentiation via phosphorylation of various signal transduction cascades^53, 54^. Therefore, we used various kinase inhibitors to examine whether melatonin is associated with phosphorylation of Osterix. As shown in Fig. 5, H89 and Go6976, which are PKA and PKC inhibitors respectively, significantly repressed Osterix-induced transcriptional activity, as well as phosphorylation of Osterix, ALP activity, and matrix mineralization, in the presence of melatonin. These results demonstrate that melatonin regulates the activity and expression of Osterix via the PKA and PKC signaling pathways. Similarly, Park *et al*. reported that melatonin enhances osteoblastic differentiation through the BMP/ERK signaling pathway^42^. In the present study, ERK inhibitor, U0126 also slightly showed inhibitory effect on melatonin induced Osterix transcriptional activity, suggesting that ERK signaling pathway could be also involved in melatonin effect on Osterix. Taken together, it is likely that the regulatory effects of melatonin on Osterix phosphorylation are related to an extensive crosstalk of various protein kinases.

The similar stimulatory effects of melatonin have been observed in chondrogenic differentiation. Melatonin has confirmed to increase glycosaminoglycan synthesis and cartilage tissue, as well as to enhance the expression of chondrogenic marker genes including aggrecan, collagen type II and X, SOX9, and BMP2 during the chondrogenic differentiation at least partially through melatonin receptors (MT) including MT1 and MT2^55^. Meanwhile, it has been reported that Osterix regulates chondrocyte differentiation and bone growth in growth plate chondrocytes during endochondral ossification^56^. Our additional results indicated that melatonin effect on the activity and expression of Osterix could be regulated by Luzindole, a melatonin receptor antagonist (Fig. S6). Considering these reports with the current study together, it could be suggested melatonin could enhance chondrocyte differentiation by up-regulation of Osterix.

**Figure 6:**
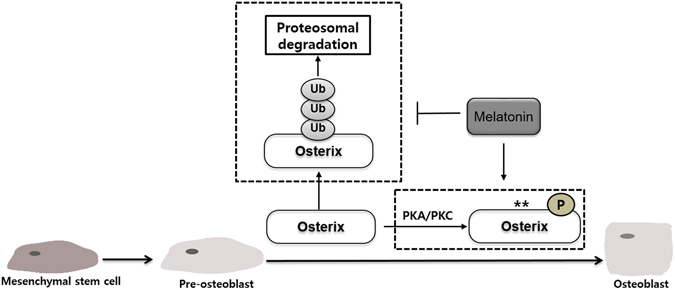
Working model for the regulatory effect melatonin in osteoblast differentiation. Melatonin stabilized the Osterix protein expression by blocking the ubiquitin-proteasome pathway and promoted osteoblast differentiation via the PKA and PKC signaling pathways.

In conclusion, the present study contributes to our understanding of melatonin function in osteoblast differentiation in several aspects. For the first time, we show that melatonin regulates Osterix expression through inhibition of the ubiquitin-proteasome system, and therefore, increases ALP activity and bone mineralization. Moreover, H89 and Go6976 reverse the effect of melatonin on Osterix-induced transactivation, suggesting that melatonin promotes osteoblast differentiation via the PKA and PKC signaling pathways (Fig. 6). Thus, these findings provide further evidence that melatonin enhances osteoblast differentiation, and hence it may be a potent osteogenic agent targeting mature osteoblast differentiation and bone formation.

## Materials and Methods

### Antibodies, plasmids, and reagents

Anti-Runx2 (ab76956; 1:1000) was purchased from Abcam (Boston, MA, USA), and anti-Osterix (A-13; 1:1000) and anit-Dlx5 (C-20; 1:1000) were purchased from Santa Cruz Biotechnology (Dallas, TX, USA). Anti-α-tubulin (B-5–1–2; 1:5000) was purchased from Sigma-Aldrich (St Louis, MO, USA). Anti-Myc (9E10; 1:1000) was purchased from Roche Applied Science. Anti-phospho-(Ser/Thr) PKA substrate (9621; 1:1000) and anti-phospho-PKC substrate motif (6967; 1:1000) were obtained from Cell Signaling Technology (Beverly, MA, USA). The plasmid for Myc-tagged Osterix was constructed in a cytomegalovirus (CMV) promoter-derived mammalian expression vector. For Osterix gene silencing, small hairpin RNA (shRNA) oligonucleotides were synthesized by targeting a 19-base pair sequence (GT CTA CAC TTC CCT GGA TA) of the mouse Osterix gene. Annealed oligonucleotides were ligated into the pSuper RNA system (Oligoengine, Seattle, WA, USA). Melatonin was purchased from Sigma-Aldrich (M5250). Cycloheximide (CHX; protein synthesis inhibitor; 239763), mitogen-activated protein kinase kinase (MEK) inhibitor U0126 (662005), protein kinase A (PKA) inhibitor H89 (371963), protein kinase C (PKC) inhibitor Go6976 (365250), SB203580 (p38 MAPK inhibitor; 559386), XI (Akt inhibitor; 124028), and glycogen synthase kinase 3 (GSK3) inhibitor LiCl (438002) were purchased from Calbiochem (San Diego, CA, USA).

### Cell culture and osteogenic differentiation

The mouse pre-myoblast cell line, C2C12, was purchased from ATCC (Rockville, MD, USA). Cells were maintained in Dulbecco’s modified Eagle’s medium (DMEM; Gibco, Waltham, MA, USA) supplemented with 10% fetal bovine serum (FBS; Welgene, Geyongsangbuk-do, Korea) and 1% antibiotic-antimycotic (Thermo Fisher Scientific, Waltham, MA, USA) at 37 °C. For induction of osteogenic differentiation, the cells were seeded and cultured until they reached confluence. The medium was then changed (day 0) to differentiation medium containing DMEM supplemented with 2% FBS and 30 ng/mL BMP-4.

### Alkaline phosphatase (ALP) and Alizarin red S (ARS) staining

Differentiated C2C12 cells were stained with the BCIP/NBT color development substrate (Sigma-Aldrich) for ALP activity. Alizarin red S (ARS) staining to evaluate calcium-rich deposits was performed as previously described^47^. Briefly, the cells were fixed in 4% formaldehyde for 15 min, then stained with 0.2% ARS (pH 7.2) solution for 30 min, and then washed twice with phosphate buffered saline (PBS). Quantification of ALP and ARS staining was performed at an absorbance of 480 and 405 nm, respectively.

### Transient transfection and luciferase reporter assay

Cells were transfected with polyethylenimine (PEI; Polysciences, Warminster, PA, USA) as previously described^57^. The total amount of transfected plasmids in each group was normalized by adding an empty vector. For luciferase reporter assay, the Osterix expression construct, the luciferase reporter (ALP-Luc, BSP-Luc, or OC-Luc), and β-galactosidase plasmid (internal control) were co-transfected into C2C12 cells. The constructs for ALP-Luc (900 bp)^58^, BSP-Luc (938 bp)^59^, and OC-Luc (1.1 kbp)^59^ were obtained as described previously. At 36 h after transfection, cell lysates were analyzed for luciferase activity. The luciferase activities were measured using Luciferase Reporter Assay kit (Promega, Madison, WI, USA), and all experiments were carried out in triplicates.

### Real-time reverse transcription (RT)-PCR

Total mRNA was extracted from cultured C2C12 cells using the RNAiso Plus (Total RNA extraction reagent; TaKaRa, Tokyo, Japan). Oligo (dT) primers and reverse transcriptase (Promega) were used to synthesize the cDNA. Real-time PCR was performed using SYBR Premix Ex Taq kit (TaKaRa) on a CFX96 real-time PCR System. Samples were incubated at 95 °C for 30 s followed by 40 cycles at 95 °C for 5 s and 60 °C for 30 s. The expression level of GAPDH (glyceraldehyde 3-phosphate dehydrogenase) was used as an internal control to normalize mRNA expression. The ΔC_t_ was determined by subtracting the C_t_ value of GAPDH from that of target. The relative expression levels of each gene were calculated by the 2^−ΔΔCt^ method. The primer sequences used for PCR were as follows: ALP: forward 5′-ATC TTT GGT CTG GCT CCC ATG-3′ and reverse 5′-TTT CCC GTT CAC CGT CCA C-3′; BSP: forward 5′-AAG CAG CAC CGT TGA GTA TGG-3′ and reverse 5′-CCT TGT AGT AGC TGT ATT CGT CCT C-3′; GAPDH: forward 5′-AGG TCG GTG TGA ACG GAT TTG-3′ and reverse 5′-GGG GTC GTT GAT GGC AAC A-3′.

### Western blotting and immunoprecipitation

Cell pellets were washed with PBS and lysed in ice-cold lysis buffer [25 mM HEPES (pH 7.5), 150 mM NaCl, 1% NP-40, 0.25% sodium deoxycholate, 10% glycerol, 25 mM NaF, 1 mM ethylenediaminetetraacetic acid (EDTA), 1 mM Na_3_VO_4_, l0 μg/mL leupeptin, and 10 μg/mL aprotinin] for 30 min, and then cleared by centrifugation at 13,200 rpm at 4 °C. Cell extracts (20 μg of protein) were separated by sodium dodecyl sulfate-polyacrylamide gel electrophoresis (SDS-PAGE) and blotted to a polyvinylidene fluoride (PVDF) membrane, blocked with 5% skim milk in Tris-buffered saline containing 0.1% Tween 20 (TBS-T). After washes with TBS-T, the membranes were incubated with designated antibodies, followed by incubation with horseradish peroxidase (HRP)-conjugated secondary antibodies. Visualization was performed by enhanced chemiluminescence using Immobilon Western Chemiluminescent HRP Substrate (Merck Millipore, Billerica, MA, USA). The band strength was quantified using the image software, Multi Gauge V3.0 (FUJIFILM, Tokyo, Japan). The same amounts of proteins were subjected to immunoprecipitation with designated antibodies and protein A agarose beads. The immunoprecipitated proteins were subjected to SDS-PAGE and visualized by immunoblotting.

### Degradation assay

C2C12 cells were cultured in 6-well plates and transfected with Myc-Osterix or pretreated with 30 ng/mL BMP-4 in the absence or presence of melatonin. Protein lysates were prepared at indicated time points after addition of CHX (40 μg/mL). Equal amounts of protein were separated by SDS–PAGE. Levels of Osterix were determined by immunoblotting and quantified at indicated time points.

### Statistical analysis

Statistical analysis was performed using GraphPad Prism 5.03 software (GraphPad Software Inc, La Jolla, CA, USA). Data were analyzed using one-way or two-way ANOVA procedure. The results are expressed as the mean ± standard deviation (SD) for the number of assays indicated. A *P* value less than 0.05 was considered significant.

## Electronic supplementary material

Supplementary figures

## References

[1] Nijweide PJ, Burger EH, Feyen JH (1986). Cells of bone: proliferation, differentiation, and hormonal regulation. Physiological reviews.

[2] Harada S, Rodan GA (2003). Control of osteoblast function and regulation of bone mass. Nature.

[3] Teitelbaum SL, Ross FP (2003). Genetic regulation of osteoclast development and function. Nature reviews. Genetics.

[4] Lin GL, Hankenson KD (2011). Integration of BMP, Wnt, and notch signaling pathways in osteoblast differentiation. Journal of cellular biochemistry.

[5] Komori T (2006). Regulation of osteoblast differentiation by transcription factors. Journal of cellular biochemistry.

[6] Lerner AB, Case JD, Mori W, Wright MR (1959). Melatonin in peripheral nerve. Nature.

[7] Reiter RJ (1991). Melatonin: the chemical expression of darkness. Molecular and cellular endocrinology.

[8] Benarroch EE (2008). Suprachiasmatic nucleus and melatonin: reciprocal interactions and clinical correlations. Neurology.

[9] Reiter RJ (1976). Melatonin inhibition of reproduction in the male hamster: its dependency on time of day of administration and on an intact and sympathetically innervated pineal gland. Neuroendocrinology.

[10] McArthur AJ, Hunt AE, Gillette MU (1997). Melatonin action and signal transduction in the rat suprachiasmatic circadian clock: activation of protein kinase C at dusk and dawn. Endocrinology.

[11] Liebmann PM, Wolfler A, Felsner P, Hofer D, Schauenstein K (1997). Melatonin and the immune system. International archives of allergy and immunology.

[12] Kvetnoi IM, Levin IM (1986). [Melatonin and tumor growth]. Eksperimental’naia onkologiia.

[13] Manchester LC (2015). Melatonin: an ancient molecule that makes oxygen metabolically tolerable. J Pineal Res.

[14] Reiter RJ (2016). Melatonin as an antioxidant: under promises but over delivers. J Pineal Res.

[15] Koyama H, Nakade O, Takada Y, Kaku T, Lau KHW (2002). Melatonin at pharmacologic doses increases bone mass by suppressing resorption through down-regulation of the RANKL-mediated osteoclast formation and activation. J Bone Miner Res.

[16] Machida M (2006). Experimental scoliosis in melatonin-deficient C57BL/6J mice without pinealectomy. Journal of pineal research.

[17] Maria S, Witt-Enderby PA (2014). Melatonin effects on bone: potential use for the prevention and treatment for osteopenia, osteoporosis, and periodontal disease and for use in bone-grafting procedures. J Pineal Res.

[18] Roth JA, Kim BG, Song F, Lin WL, Cho MI (1999). Melatonin promotes osteoblast differentiation and bone formation (vol 274, pg 22041, 1999). Journal of Biological Chemistry.

[19] Satomura K (2007). Melatonin at pharmacological doses enhances human osteoblastic differentiation *in vitro* and promotes mouse cortical bone formation *in vivo*. Journal of pineal research.

[20] Son JH (2014). Melatonin promotes osteoblast differentiation and mineralization of MC3T3-E1 cells under hypoxic conditions through activation of PKD/p38 pathways. J Pineal Res.

[21] Choi YH, Gu YM, Oh JW, Lee KY (2011). Osterix is regulated by Erk1/2 during osteoblast differentiation. Biochem Bioph Res Co.

[22] He S (2014). Protein Kinase A Regulates the Osteogenic Activity of Osterix. J Cell Biochem.

[23] Wang XY, Goh CH, Li BJ (2007). p38 Mitogen-activated protein kinase regulates osteoblast differentiation through Osterix. Endocrinology.

[24] Choi YH (2011). Akt phosphorylates and regulates the osteogenic activity of Osterix. Biochem Bioph Res Co.

[25] Xu YX (2015). Phosphorylation of Serine422 increases the stability and transactivation activities of human Osterix. Febs Lett.

[26] Witt-Enderby PA, Radio NM, Doctor JS, Davis VL (2006). Therapeutic treatments potentially mediated by melatonin receptors: potential clinical uses in the prevention of osteoporosis, cancer and as an adjuvant therapy. J Pineal Res.

[27] Sanchez-Barcelo EJ, Mediavilla MD, Tan DX, Reiter RJ (2010). Scientific basis for the potential use of melatonin in bone diseases: osteoporosis and adolescent idiopathic scoliosis. Journal of osteoporosis.

[28] Sack RL, Lewy AJ, Erb DL, Vollmer WM, Singer CM (1986). Human melatonin production decreases with age. J Pineal Res.

[29] Ladizesky MG (2001). Effect of melatonin on bone metabolism in ovariectomized rats. Life sciences.

[30] Walecka-Kapica E (2015). Melatonin and female hormone secretion in postmenopausal overweight women. International journal of molecular sciences.

[31] Amstrup AK, Sikjaer T, Heickendorff L, Mosekilde L, Rejnmark L (2015). Melatonin improves bone mineral density at the femoral neck in postmenopausal women with osteopenia: a randomized controlled trial. J Pineal Res.

[32] Cutando A, Gomez-Moreno G, Arana C, Acuna-Castroviejo D, Reiter RJ (2007). Melatonin: potential functions in the oral cavity. J Periodontol.

[33] Roth JA, Kim BG, Lin WL, Cho MI (1999). Melatonin promotes osteoblast differentiation and bone formation. The Journal of biological chemistry.

[34] Sethi S (2010). Determination of the minimal melatonin exposure required to induce osteoblast differentiation from human mesenchymal stem cells and these effects on downstream signaling pathways. J Pineal Res.

[35] Radio NM, Doctor JS, Witt-Enderby PA (2006). Melatonin enhances alkaline phosphatase activity in differentiating human adult mesenchymal stem cells grown in osteogenic medium via MT2 melatonin receptors and the MEK/ERK (1/2) signaling cascade. J Pineal Res.

[36] Son JH (2014). Melatonin promotes osteoblast differentiation and mineralization of MC3T3-E1 cells under hypoxic conditions through activation of PKD/p38 pathways. J Pineal Res.

[37] Zhang L (2010). Melatonin inhibits adipogenesis and enhances osteogenesis of human mesenchymal stem cells by suppressing PPARgamma expression and enhancing Runx2 expression. J Pineal Res.

[38] Zhang C (2010). Transcriptional regulation of bone formation by the osteoblast-specific transcription factor Osx. Journal of orthopaedic surgery and research.

[39] Nakashima K (2002). The novel zinc finger-containing transcription factor Osterix is required for osteoblast differentiation and bone formation. Cell.

[40] Chen G, Deng C, Li YP (2012). TGF-beta and BMP signaling in osteoblast differentiation and bone formation. International journal of biological sciences.

[41] Ulsamer A (2008). BMP-2 induces osterix expression through up-regulation of Dlx5 and its phosphorylation by p38. Journal of Biological Chemistry.

[42] Park KH (2011). Melatonin promotes osteoblastic differentiation through the BMP/ERK/Wnt signaling pathways. J Pineal Res.

[43] Ladizesky MG (2003). Melatonin increases oestradiol-induced bone formation in ovariectomized rats. J Pineal Res.

[44] Peng, Y. Y. *et al*. Characterization of Osterix Protein Stability and Physiological Role in Osteoblast Differentiation. *Plos One* **8**, doi:10.1371/journal.pone.0056451 (2013). 10.1371/journal.pone.0056451 PMC3574093 23457570

[45] Vriend J, Reiter RJ (2014). Melatonin and ubiquitin: what’s the connection?. Cell Mol Life Sci.

[46] Vriend J, Reiter RJ (2016). Melatonin, bone regulation and the ubiquitin-proteasome connection: A review. Life sciences.

[47] Choi YH (2015). Cbl-b and c-Cbl negatively regulate osteoblast differentiation by enhancing ubiquitination and degradation of Osterix. Bone.

[48] Lian C (2016). Melatonin reversed tumor necrosis factor-alpha-inhibited osteogenesis of human mesenchymal stem cells by stabilizing SMAD1 protein. J Pineal Res.

[49] Ortuno MJ (2010). p38 regulates expression of osteoblast-specific genes by phosphorylation of osterix. The Journal of biological chemistry.

[50] Xu Y (2015). Phosphorylation of Serine422 increases the stability and transactivation activities of human Osterix. Febs Lett.

[51] Choi YH (2015). Src enhances osteogenic differentiation through phosphorylation of Osterix. Mol Cell Endocrinol.

[52] Nguyen, L. K., Kolch, W. & Kholodenko, B. N. When ubiquitination meets phosphorylation: a systems biology perspective of EGFR/MAPK signalling. *Cell Commun Signal* **11**, doi:10.1186/1478-811x-11-52 (2013). 10.1186/1478-811X-11-52 PMC3734146 23902637

[53] Sethi S (2011). Determination of the minimal melatonin exposure required to induce osteoblast differentiation from human mesenchymal stem cells and these effects on downstream signaling pathways (vol 49, pg 222, 2010). J Pineal Res.

[54] Letellier K (2008). Estrogen cross-talk with the melatonin signaling pathway in human osteoblasts derived from adolescent idiopathic scoliosis patients. J Pineal Res.

[55] Gao WJ (2014). Melatonin enhances chondrogenic differentiation of human mesenchymal stem cells. J Pineal Res.

[56] Oh JH, Park SY, de Crombrugghe B, Kim JE (2012). Chondrocyte-specific ablation of Osterix leads to impaired endochondral ossification. Biochem Bioph Res Co.

[57] Longo PA, Kavran JM, Kim MS, Leahy DJ (2013). Transient mammalian cell transfection with polyethylenimine (PEI). Methods in enzymology.

[58] Kim YJ, Lee MH, Wozney JM, Cho JY, Ryoo HM (2004). Bone morphogenetic protein-2-induced alkaline phosphatase expression is stimulated by Dlx5 and repressed by Msx2. Journal of Biological Chemistry.

[59] Gordon JAR (2010). Pbx1 Represses Osteoblastogenesis by Blocking Hoxa10-Mediated Recruitment of Chromatin Remodeling Factors. Molecular and cellular biology.

