# Supplementary material for: Melatonin promotes osteoblast differentiation by regulating Osterix protein stability and expression
Source: Sci Rep. 2017 Jul 18;7:5716. doi: 10.1038/s41598-017-06304-x (PMC5515917; doi:10.1038/s41598-017-06304-x)
Supplement: Supplementary file 1 — Supplementary figures [file 41598_2017_6304_MOESM1_ESM.pdf]

Melatonin promotes osteoblast differentiation by regulating Osterix protein stability and expression

Younho Han[1], Young-Mi Kim[2], Hyung Sik Kim[3] and Kwang Youl Lee [1,*]

SUPPLEMENTARY INFORMATION

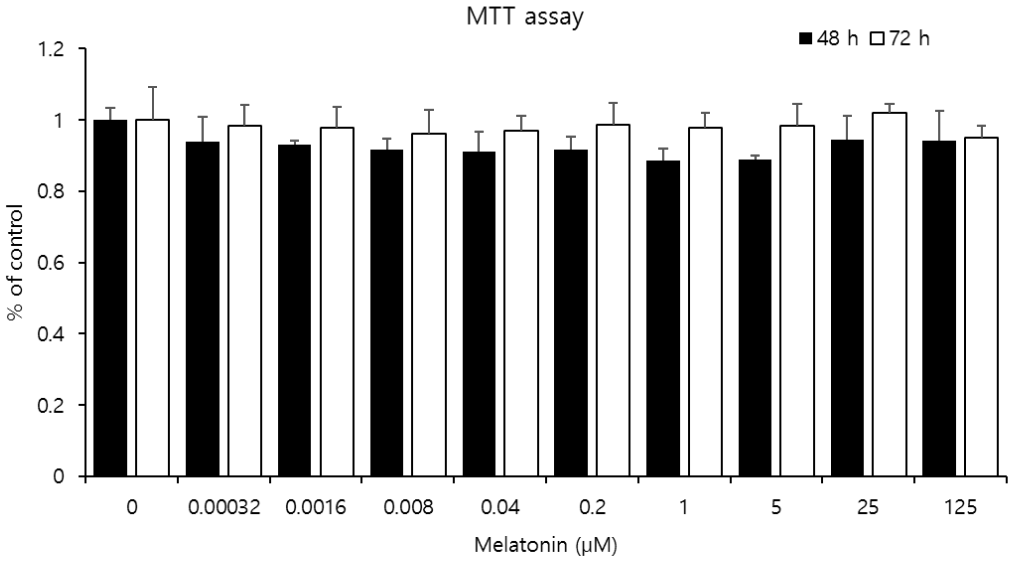

Fig S1. The effect of melatonin on the cell proliferation of C2C12. Adherent cells that proliferated in 96-well plates were incubated with different concentrations of melatonin for 48 h or 72 h. Cell proliferation was determined by MTT assay.

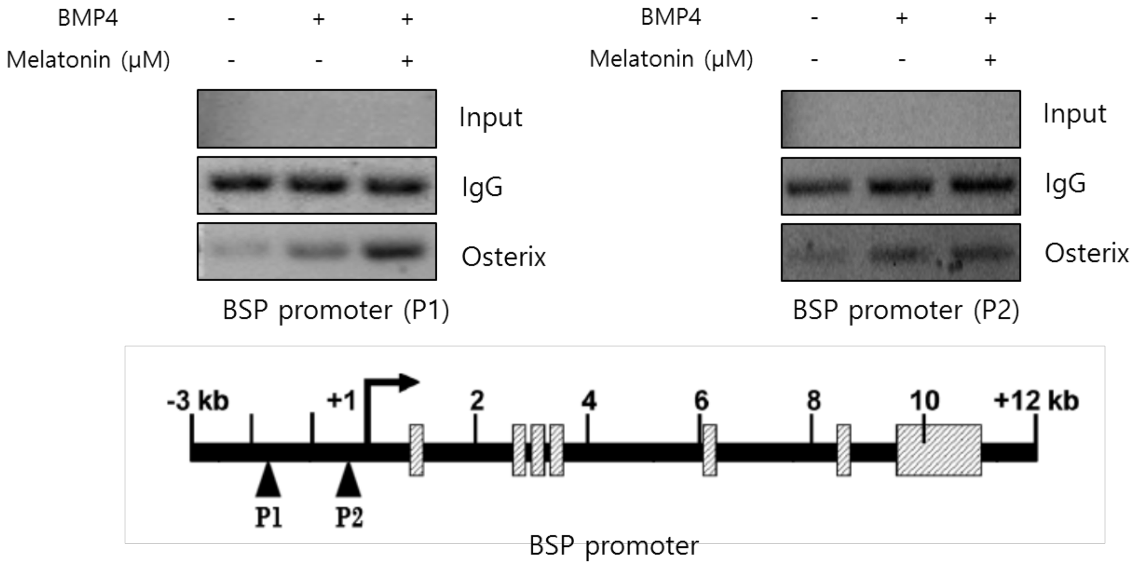

Fig. S2. Melatonin enhances the binding of Osx on the BSP promoter. ChIP assay was performed on C2C12 cells chromatin after BMP4 induction on the stage of day 3 using anti-Osx or anti-IgG antibody. The immunoprecipitated DNA was used as a PCR template to detect the two BSP promoter regions (P1, and P2).

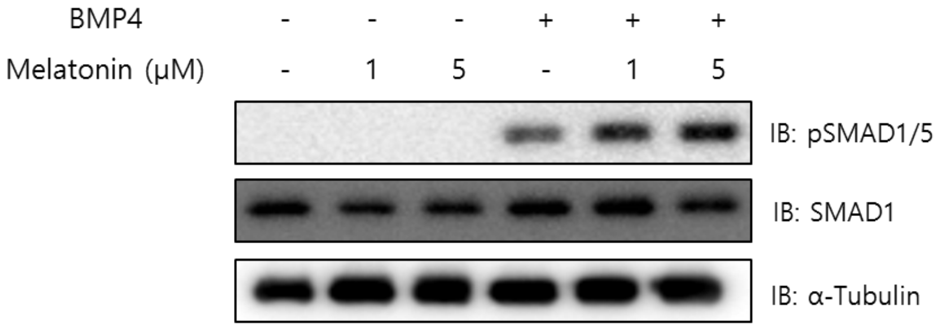

Fig S3. The effect of melatonin BMP4-induced SMAD signaling. C2C12 cells were pretreated with indicated concentrations of melatonin for 24 h and BMP-4 (30 ng/mL) exposed for 30 min. Protein levels of pSMAD1/5 and SMAD1 were detected using immunoblotting. α-tubulin was used as a loading control.

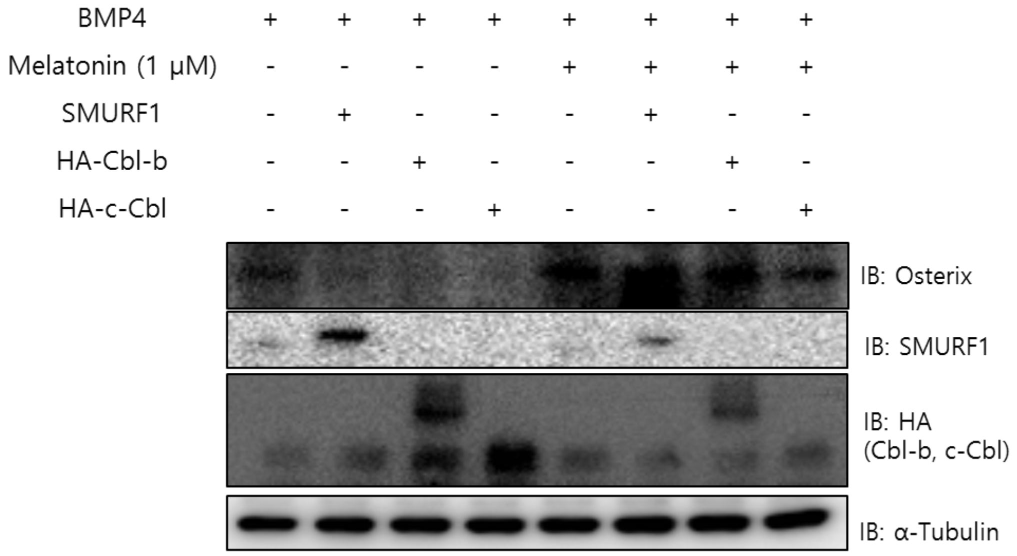

Fig S4. The recovery effect of melatonin on E3 ligase induced down-regulation of Osterix expression. C2C12 cells were transfected with indicated E3 ubiquitin ligases including SMURF1, Cbl-b, and c-Cbl and then treated with BMP-4 (30 ng/mL) and exposed to melatonin (1 μM) for 3 days. Protein levels of Osterix and indicated E3 ubiquitin ligases were detected using immunoblotting. α-tubulin was used as a loading control.

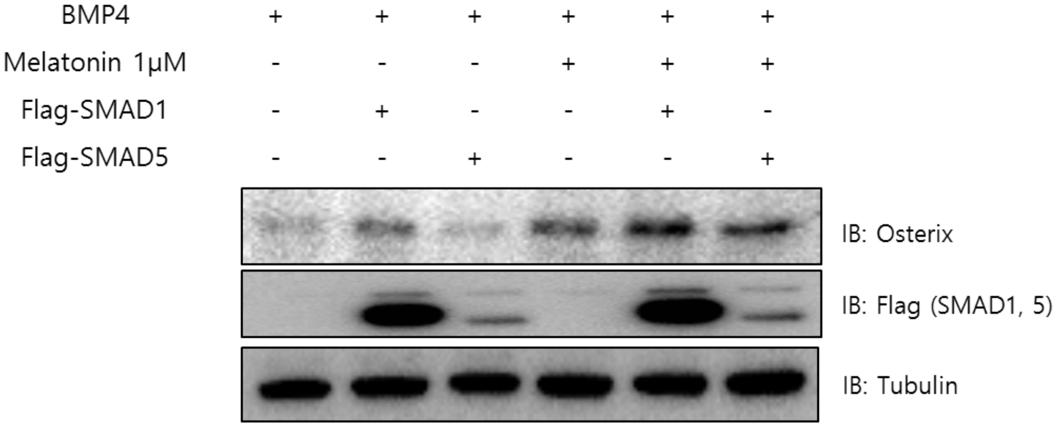

Fig S5. The stimulatory effect of melatonin on SMAD1/5 mediated up-regulation of Osterix expression. C2C12 cells were transfected with indicated SMADs (Flag-SMAD1 or Flag-SMAD5) and then treated with BMP-4 (30 ng/mL) and exposed to melatonin (1 μM) for 3 days. Protein levels of Osterix and SMADs were detected using immunoblotting. α-tubulin was used as a loading control.

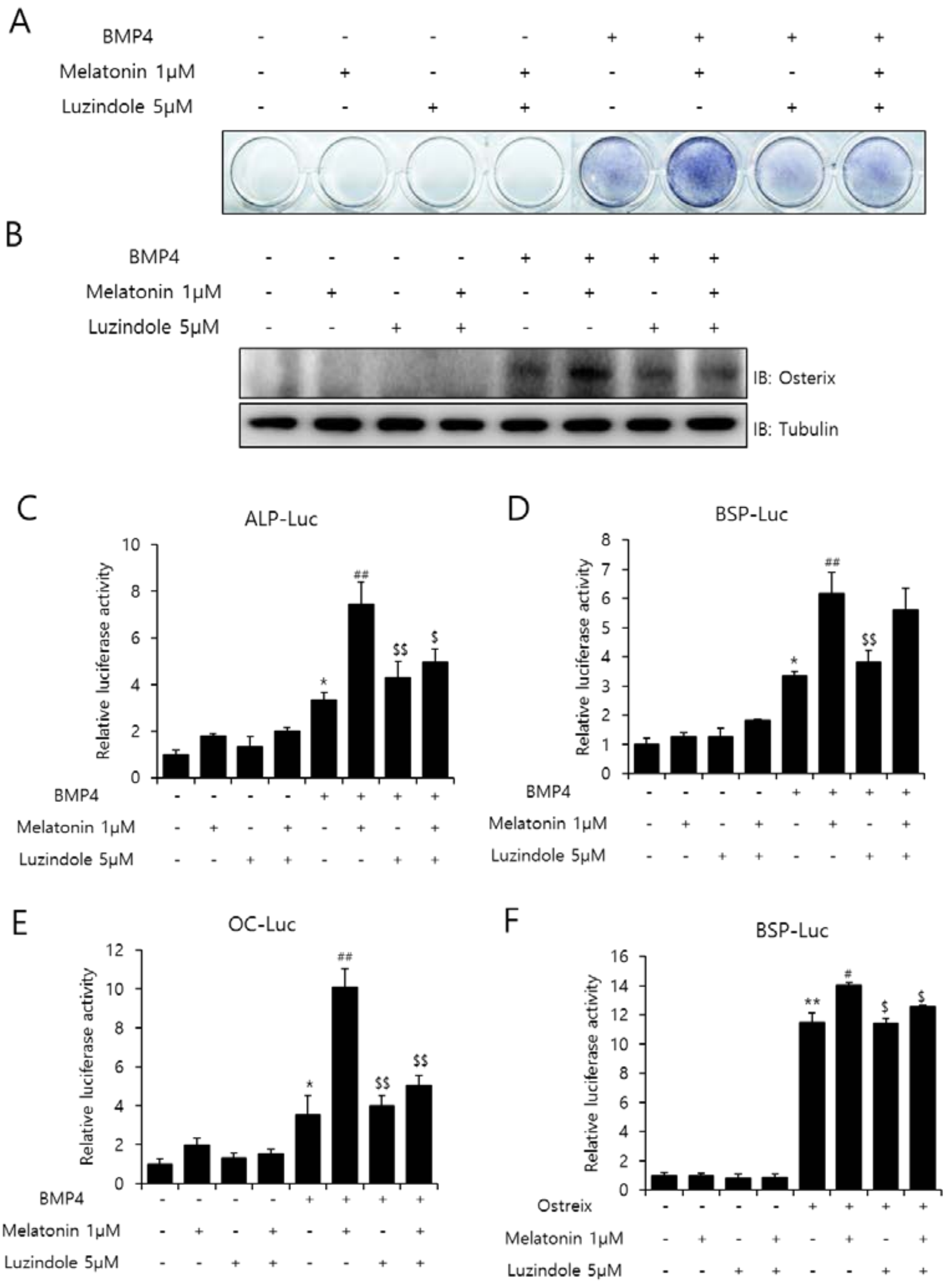

Fig S6. The inhibitory effect of luzindole on the activity and expression of Osterix induced by melatonin. (A) C2C12 cells were treated with BMP-4 (30 ng/mL) and exposed to melatonin (1μM) and luzindole (5μM) for 3 days (ALP staining). (B) C2C12 cells were treated with BMP-4 (30 ng/mL) and exposed to melatonin (1 μM) and luzindole (5μM) for 3 days. Protein levels of Osterix was detected using immunoblotting. α-tubulin was used as a loading control. (C-E) C2C12 cells were transfected with pCMV-β-gal (0.1 μg), luciferase reporters [(C) ALP-Luc, (D) BSP-Luc, or (E) OC-Luc; 0.3 μg] and exposed to indicated combinations of melatonin (1 μM) and luzindole (5μM). Luciferase activities were measured. (F) C2C12 cells were transfected with pCMV-β-gal (0.1 μg), Myc-Osterix (0.3 μg), and luciferase reporter (BSP-Luc, 0.3 μg), and exposed to indicated combinations of melatonin (1 μM) and luzindole (5μM). Luciferase activities were measured. *$*P < 0.05$, $**P < 0.01$*

compared with control group. $^{\#}P < 0.05$, $^{\#\#}P < 0.01$ compared with BMP-4-treated group. $^{\$}P < 0.05$, $^{\$\$}P < 0.01$ compared with BMP-4- and melatonin-treated group. Statistical analysis by one-way ANOVA Data are representative of three independent experiments [mean ± SD of two replicates in A and B and three replicates in C-F].

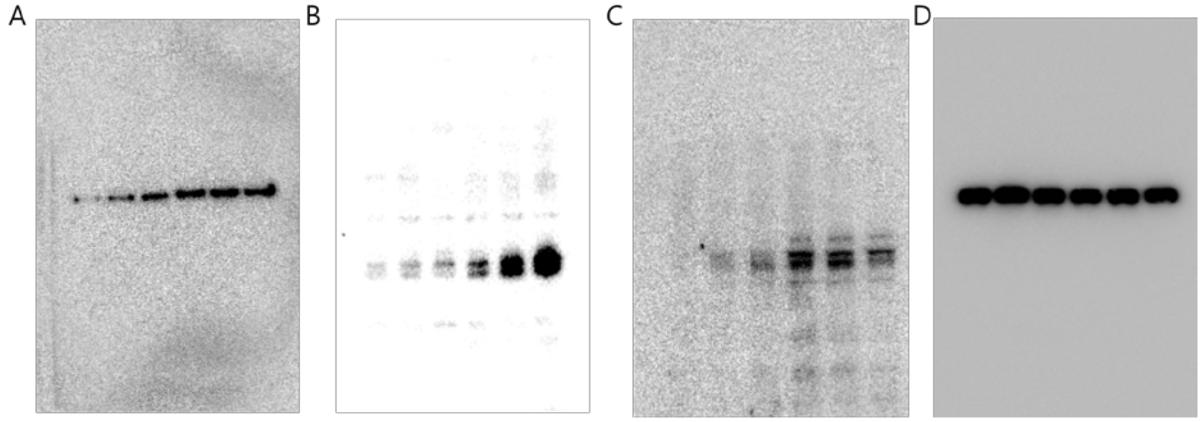

Fig S7. High resolution of full-length membrane blots corresponding to Fig. 1D (A) IB: Runx2. (B) IB: Osterix. (C) IB: Dlx5 (D) IB: α-Tubulin.

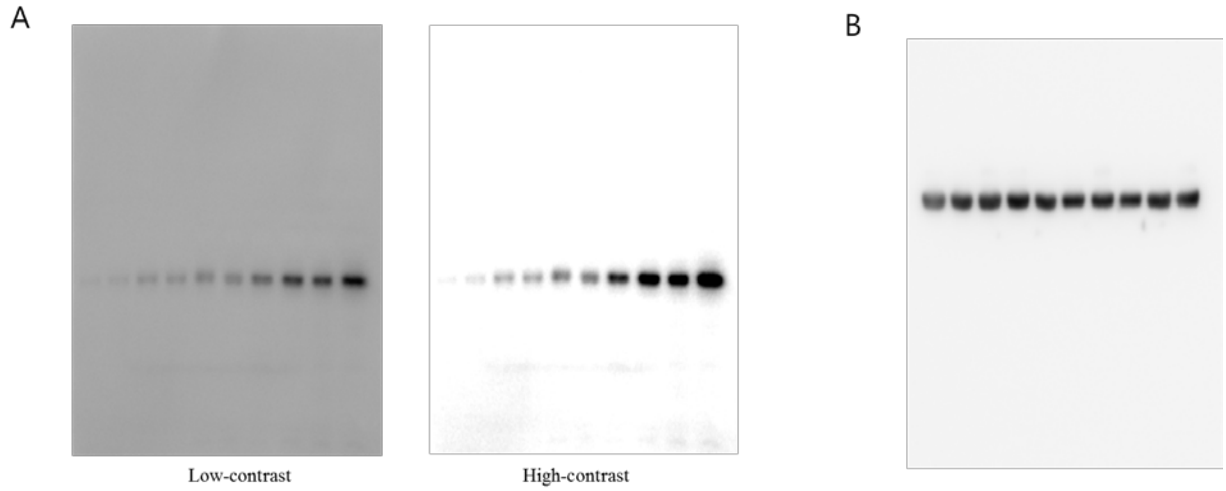

Fig S8. High resolution of full-length membrane blots corresponding to Fig. 3A (A) IB: Osterix. (B) IB: α-Tubulin.

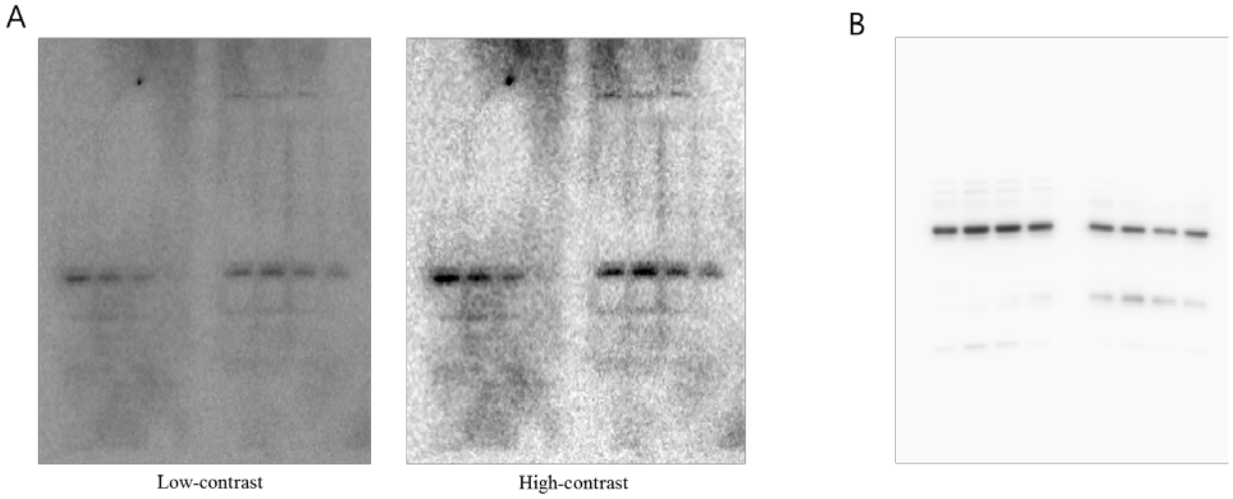

Fig S9. High resolution of full-length membrane blots corresponding to Fig. 3B (A) IB: Myc (Osterix). (B) IB: α-Tubulin.

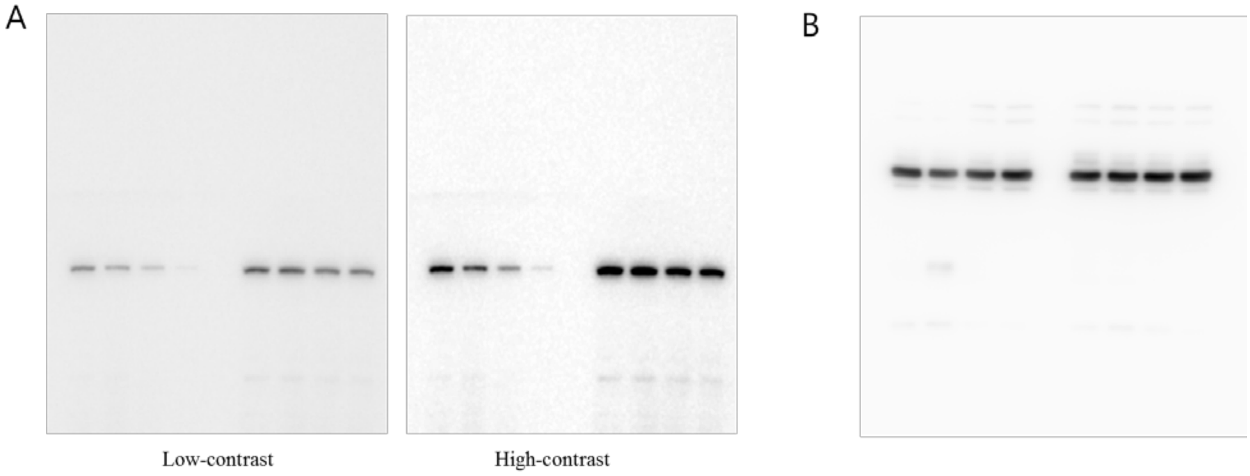

Fig S10. High resolution of full-length membrane blots corresponding to Fig. 3C (A) IB: Osterix. (B) IB: α-Tubulin.

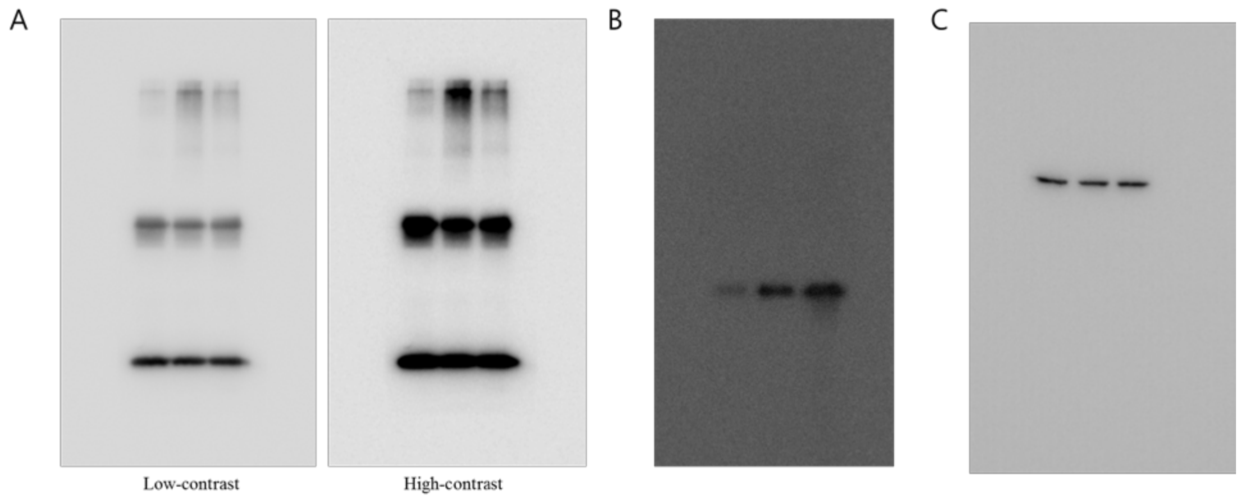

Fig S11. High resolution of full-length membrane blots corresponding to Fig. 3D (A) IP: Osterix, IB: Ubiquitin. (B) IB: Osterix. (C) IB: α-Tubulin.

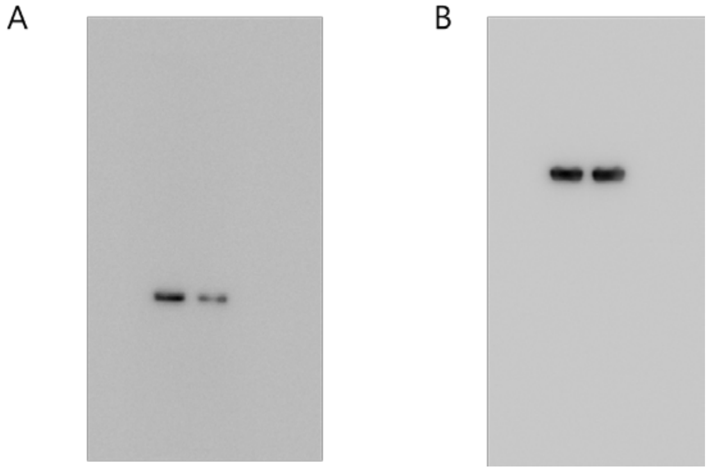

Fig S12. High resolution of full-length membrane blots corresponding to Fig. 4E (A) IB: Osterix. (B) IB: α-Tubulin.

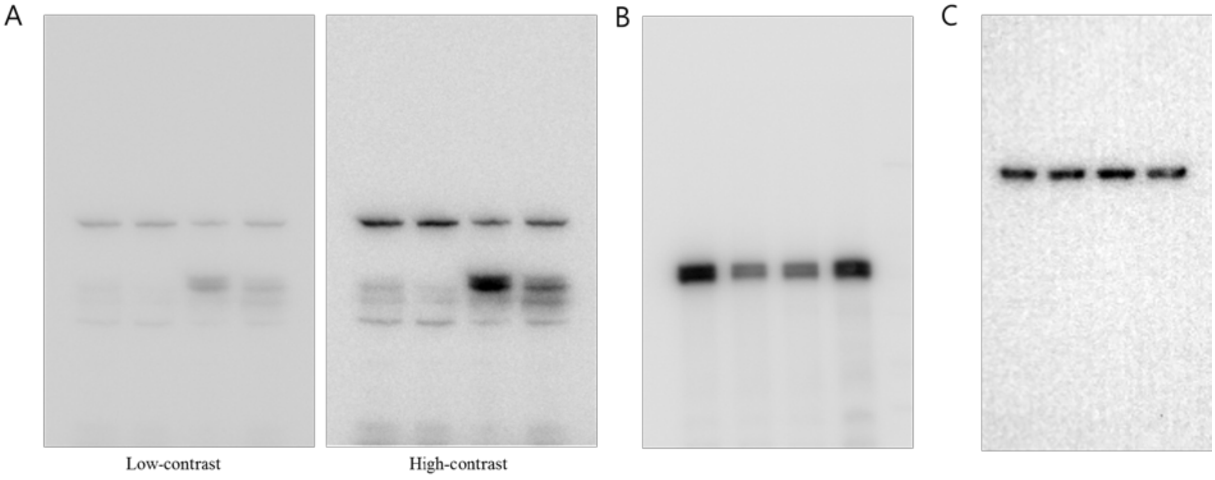

Fig S13. High resolution of full-length membrane blots corresponding to Fig. 5D (A) IP: p-PKA sub. IB: Osterix (B) IB: Osterix. (C) IB: α-Tubulin.

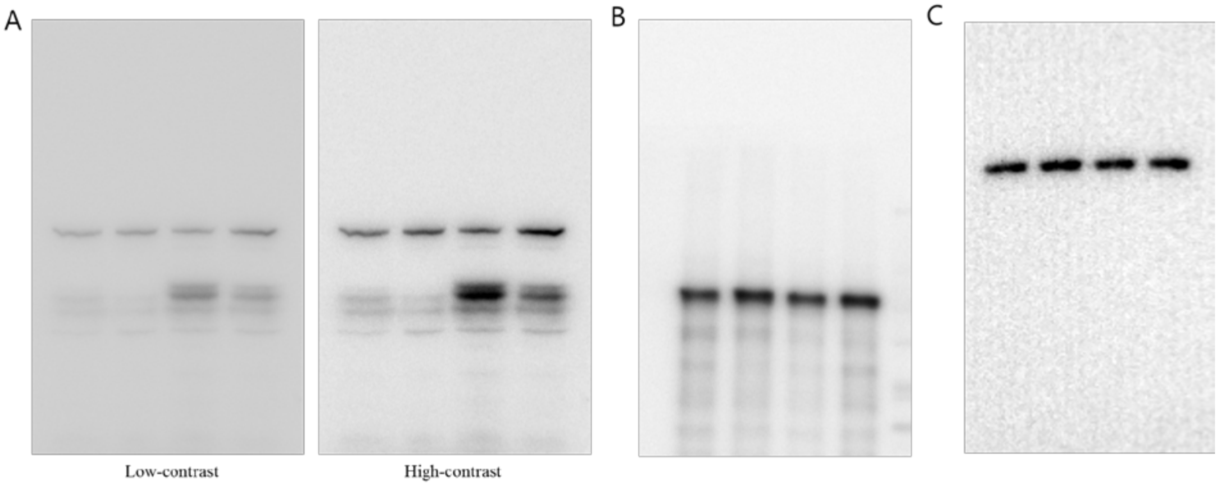

Fig S14. High resolution of full-length membrane blots corresponding to Fig. 5E (A) IP: p-PKC sub. IB: Osterix (B) IB: Osterix. (C) IB: α-Tubulin**.**
